# The Impacts of* Chrysanthemum indicum* Extract on Oxidative Stress and Inflammatory Responses in Adjuvant-Induced Arthritic Rats

**DOI:** 10.1155/2017/3285394

**Published:** 2017-04-10

**Authors:** Mei Dong, Dongsheng Yu, Naif Abdullah Al-Dhabi, Veeramuthu Duraipandiyan

**Affiliations:** ^1^Infectious Immune Department of Rheumatism, Tianjin Hospital, Jiefangnan Road 406, Tianjin 300211, China; ^2^Department of Rehabilitation Medicine, Tianjin Medical University, Tianjin 300070, China; ^3^Addiriyah Chair for Environmental Studies, Department of Botany and Microbiology, College of Science, King Saud University, P.O. Box 2455, Riyadh 11451, Saudi Arabia

## Abstract

*Chrysanthemum indicum* has been used as a therapeutic agent against inflammation, hypertension, and respiratory conditions for many years. This research's aim has been to examine the antioxidant impacts that* Chrysanthemum indicum* extract (CIE) has on the oxidative stress and inflammatory responses in adjuvant-induced arthritic (AA) rats. 40 rats were categorised into 4 groups according to a completely randomized approach: Group I involved normal control rats (CTRL) that received a basal diet; Group II involved arthritic control rats (CTRL-AA) that received the same diet; Group III involved rats that received a basal diet and 30 mg/kg CIE; and Group IV involved arthritic rats with the same diet as Group III rats (CIE-AA). After injection with complete Freund's adjuvant, body weight, arthritis score, and the serum levels of TNF-*α*, IL-1*β*, IL-6, myeloperoxidase (MPO), malondialdehyde (MDA), superoxide dismutase (SOD), and glutathione peroxidase (GSH-PX) were assessed. The results demonstrated that CIE delayed the onset time of arthritis and decreased the clinical arthritis severity score (*P* < 0.05). Observations of CIE-AA and CTRL-AA rats demonstrated that CIE alleviates oxidative stress and inflammatory responses in CIE-AA group. In conclusion, CIE alleviated oxidative stress and inflammatory responses, thereby highlighting its potential use as a candidate for clinical treatments of rheumatoid arthritis.

## 1. Introduction

The cardinal symptoms of rheumatoid arthritis (RA), an autoimmune disease, are chronic synovitis and the impairment of articular cartilage and the underlying bone in joints. It is classified as a systemic inflammatory disease which targets the joints by generating proliferative synovitis. Over time, RA has the potential to lead to the malformation or destruction of affected joints, and this has been found to lead to working disability and higher mortality rates [1]. Research shows that approximately 1% of the global population suffers from the condition, and it has been identified in patients ranging from 35 to 50 [1].

Disequilibrium regarding pro- and anti-inflammatory cytokines has been found to initiate autoimmunity and lasting inflammation, and this is the factor that contributes to the RA's characteristic joint impairment [2]. After observing the way in which joint destruction and levels of proinflammatory cytokines in the serum or arthritic tissues of RA patients are positively related, researchers identified that a range of proinflammatory cytokines, including tumour necrosis factor- (TNF-) *α*, interleukin- (IL-) 1*β*, and IL-6, perform a significant function in the condition's biological process [3].

For transgenic mice displaying the overexpression of TNF-*α*, acute inflammatory responses and the rapid onset of destructive arthritis are consistently observed [4]. In contrast to this, IL-1 deficient mice [5] or IL-6 deficient mice [6], in the context of experimental animal models of human RA, display reduced synovial infiltrate and tissue impairment. It is worthwhile to note the evidence to suggest that biologic medications for TNF-*α*, IL-1, and IL-6 lower the radiographic onset of joint disease at the same time as they hinder the condition's activity [7]. Also notable are the findings demonstrating that the nuclear factor-*κ*B (NF-*κ*B), which is primarily constituted of p65 and p50 complex, performs an important function in transcriptionally regulating proinflammatory gene expression in the context of RA [8]. Furthermore, it is necessary for the inhibitor of NF-*κ*B (I*κ*B)*α* to degrade if NF-*κ*B is to be activated, and this drives the nuclear transport of NF-*κ*Bp65 [9]. The regulation of cytokine gene expression takes place based on NF-*κ*B activation, and in contrast to this, appropriate receptors can be drawn on by cytokines to facilitate I*κ*B*α* degradation and NF-*κ*B activation. The consequence of this is the enhancement of RA's inflammation development. An important finding to consider for the present study is the way in which significant oxidative stress, in a similar way to the rise of proinflammatory cytokines, functions as a central risk factor for joint damage in RA. The stimulation of inflammatory cells, including neutrophils and macrophages, to discharge reactive oxygen species (ROS) in the synovial fluid takes place in view of cytokine overproduction, the relevance of which is emphasised insofar as this serves as an intermediary of tissue damage [10].

Contemporary clinical practice primarily draws on disease-modifying antirheumatic drugs, among other means, for RA treatment. Other commonly employed agents range from nonsteroidal anti-inflammatory drugs and corticosteroids to biologic medications. In view of this, it is important to acknowledge that several damaging secondary effects result from the use of many of these approaches, the most notable of which include ruptured gastrointestinal blood vessels, cardiovascular complications, and liver conditions [11, 12]. Given the commonality of these side effects, survey evidence has been published to suggest that 60–90% of individuals receiving these treatments look for supplementary or substitute therapies [13].

Innovative medications sourced from curative plants have historically offered significant treatment options for various conditions, including RA. Consequently, researchers have taken as their subject the attempt to identify botanically derived drugs. The inflorescence or bud of* Chrysanthemum indicum* has found extensive usage throughout the historical practice of TCM, and it has primarily been applied in treating inflammation, hypertension, and respiratory diseases. Phytochemical profile of CIE has identified flavonoids, terpenoids, and phenolic compounds [14], and other studies have published findings to highlight its antiviral, antioxidant, anti-inflammatory, antibacterial, and immunomodulatory characteristics [15]. Given the organic nature of the therapeutic agent in combination with the widespread usage it enjoys in traditional medicine and the culinary sphere,* Chrysanthemum indicum* constitutes a promising candidate for alternative medical practice, particularly regarding the alleviation of RA's symptoms and other organ manifestations.

Therefore, it is important to account for the gap in the literature with regard to the matter of investigating the anti-inflammatory and immunomodulatory features of the plant's active components, and this constitutes the primary intention of this study. Specifically, the author will examine the impact that CIE has on paw swelling, joint impairment, the generation of inflammatory mediators, and NF-*κ*B activation in adjuvant arthritis (AA) rats.

## 2. Materials and Methods

### 2.1. *Chrysanthemum indicum* Extract Preparation

After gathering* Chrysanthemum indicum* Linné (Asteraceae) flowers at a nearby market, authentication was conducted by examining microscopic and macroscopic features. 70% ethanol (with a 2-hour reflux) was used to extract the* Chrysanthemum indicum*'s dried flowers two times, and a reduced pressure was subsequently used to concentrate the extract. Prior to storing the concentrated extract at 4°C, it was subject to filtering and lyophilization. The dried extract's yield from the initial resources equaled 12.35%. Then the lyophilized powder was suspended in 10% dimethyl sulfoxide (DMSO) to lyse the cells, filtered with a 0.2 *μ*m syringe filter, and subsequently lyophilized.

### 2.2. Laboratory Animals and Adjuvant Arthritis

After obtaining 40 2-month-old adult male Wistar rats weighing between 180 and 200 g from the Tianjin Laboratory Animal Centre (Tianjin, China), conventional environmental conditions were used for maintenance: namely, a 12-hour light/dark cycle, 25 ± 2°C, and 50% humidity. Food and drinking water were freely available for the animals. The research protocol received approval based on Tianjin Hospital's regulatory requirements for the care and use of experimental animals, and the experiment was conducted in accordance with relevant provisions.

A completely randomized approach was used to allocate 10 rats to one of 4 groups. The features of each group are listed as follows: the first group (Group I) involved normal control rats (CTRL) managed with a basal diet; the second group (Group II) involved arthritic control rats (CTRL-AA) managed with the same diet; the third group (Group III) involved rats managed with a basal diet and 30 mg/kg CIE; and the fourth group (Group IV) involved arthritic rats managed with the same diet as Group III rats (CIE-AA).

These distinct diets were maintained for each group for a period of 7 days and, following this, the arthritic rats in the CTRL-AA and CIE-AA groups were subject to anaesthetisation using isoflurane. Arthritis was brought about with one intradermal injection of 4 mg heat-killed* Mycobacterium butyricum* in Freud's adjuvant with 0.1 ml of paraffin oil. With 7-day intervals, the body weight was logged three times from day 0 to day 14 following induction by injection. The mice were sacrificed after the treatment process had finished on day 14, and then the arthritis index for each specimen was evaluated by examining the paws. The evaluation scale ranged from 0 to 4, where 0 was equivalent to no erythema or swelling; 1 to moderate erythema or swelling of a single or multiple digits; 2 to a wholly swollen paw; 3 to erythema and ankle swelling; and 4 to ankylosis (namely, the inability for ankle bending). A severity score was derived as the composite of the sum of each paw's score. Day 14 also involved the extraction of tissues for homogenate preparation from joint, and after extraction, the tissues were subject to immediate freezing and storage at −80°C for further analysis.

### 2.3. Measurement of Serum Indicators

ELISA determination kits were employed to identify the levels of TNF-*α*, IL-1*β*, and IL-6, and this was carried out based on the conventional curve (Beyotime Institute of Biotechnology, China) [16]. A Bio-Rad microplate reader (Bio-Rad Laboratories, Inc., Hercules, CA, USA) was used to log the optical density at 405 nm, and the process articulated by Liu et al. [17] was conducted to analyse myeloperoxidase (MPO), malondialdehyde (MDA), superoxide dismutase (SOD), and glutathione peroxidase (GSH-PX) activity.

### 2.4. Preparation of Whole Cell Extract for NF-*κ*B Determination

Following the experimental process, 10 mg joint tissue samples were extracted. Incubation then took place with a 100 *μ*L tissue lysis buffer (Thomas Scientific, Swedesboro, NJ, USA) for a duration of 30 minutes on ice. A BCA kit (Bio-Rad Laboratories, Inc.) was used to assess the protein concentration, and a TransAM NF-*κ*B p65 Transcription Factor Assay Kit facilitated the monitoring of NF-*κ*B activation. Quantity One software, version 4.4.0 (Bio-Rad Laboratories, Inc.), was employed to measure absorbance, and this was identified as 450 nm. The recorded outcomes were articulated in the form of absorbance per milligram of total protein.

### 2.5. Statistical Analysis

SPSS 21.0 for Windows was used to facilitate data analysis with a nonparametric Mann–Whitney test. For each of the four groups, the researcher carried out a one-way analysis of variance, and the results were articulated in the form of mean ± standard error of the mean (SEM). Intergroup comparative analysis was facilitated by employing the post hoc least squared differences (LSD) test with *P* < 0.05 being regarded as statistically significant.

## 3. Results

**Figure 1:**
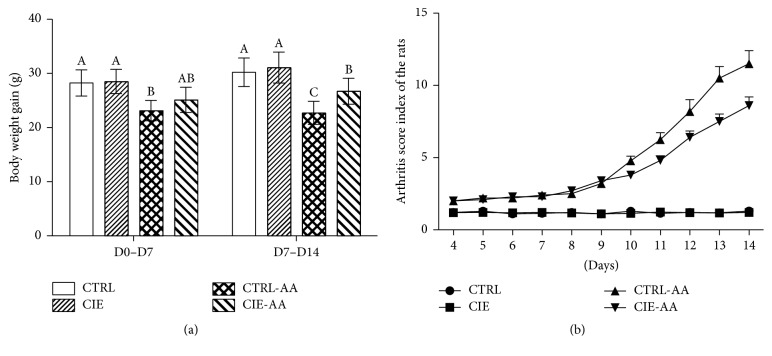
Body weight gain and arthritis index scores for Groups I–IV. (a) Body weight gain on day 7 and day 14; (b) arthritis index score from day 0 to day 14 following adjuvant injection. Arthritis was found to lower body weight gain while IF-AA treatment facilitated a rise in body weight gain when considered in relation to the CTRL-AA group. Data are expressed as means ± SEM (*n* = 10). ^A,B^Different from each other (*P* < 0.05).

Figure 1(a) presents the observed increase in body weight over the course of the experiment, and it shows that the weight of the arthritic control rats decreased significantly while the normal control rats' body weight increased (*P* < 0.05). On day 7, the recorded body weight increase for the CTRL-AA group was considerably lower when compared to the CTRL and CIE groups (*P* < 0.05), and the body weight increase for the CIE-AA group was not significantly different than the other three. On day 14, the recorded body weight increase for the CTRL-AA and CIE-AA groups was considerably lower when compared to the other two groups (*P* < 0.05), while the body weight for the CIE-AA group was considerably lower than the CTRL-AA group (*P* < 0.05, see Figure 1(a)).  Figure 1(b) displays the progression of the arthritis score index. Over the course of the initial phase of the condition (namely, until the eighth day following adjuvant injection), an examination of the arthritic rats revealed a minor inflammatory reaction in the injected paw; the arthritis scores for the injected paws ranged between 2 and 3 for the CTRL-AA and CIE-AA groups. Inflammation was seen to commence on day 9, and for the CTRL-AA group, arthritis scores rose to the highest end of the scale on day 14. For the CTRL-AA and CIE-AA groups, the arthritis scores were notably greater than those of the other two groups from day 7 (*P* < 0.05), and the CTRL-AA group's arthritis sores were notably greater than that of the CIE-AA group at day 10 (*P* < 0.05).

**Figure 2:**
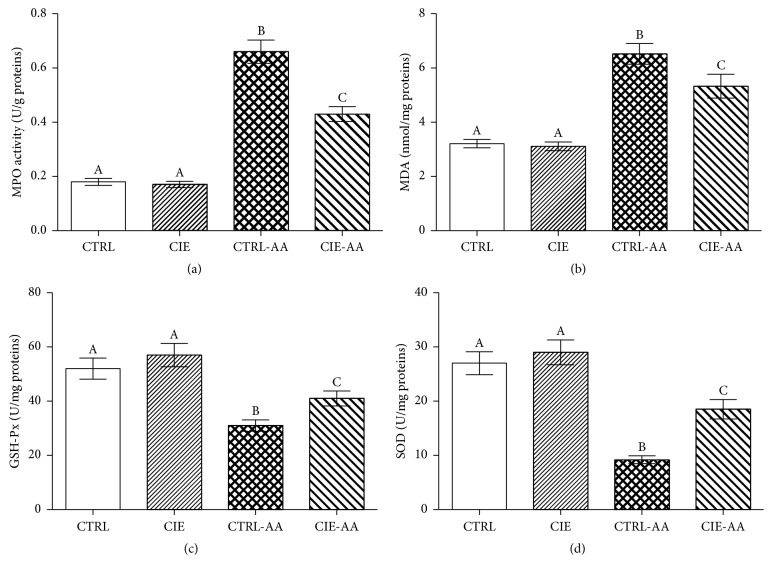
The preventative impact of CIE on the level of (a) MPO, (b) MDA, (c) GSH-Px, and (d) SOD values for the AA model. Data indicated with different superscript letters were statistically dissimilar (*P* < 0.05; *n* = 10).

As demonstrated in Figures 2(a) and 2(b), when considering the CTRL and CIE groups in relation to the CTRL-AA and CIE-AA groups, the MPO and MDA levels in the serum were significantly higher for the latter (*P* < 0.05). Additionally, the CIE-AA group displayed notably lower MPO and MDA levels (*P* < 0.05) when considered in relation to the CTRL-AA group (Figures 2(a) and 2(b)). As seen in Figures 2(c) and 2(d), a significant inhibition in the level of GSH-Px and SOD (*P* < 0.05) in the CTRL-AA and CIE-AA groups was observed by way of comparison with the CTRL and CIE groups. Furthermore, when comparing the CIE-AA group with the CTRL-AA group, the activity of GSH-Px and SOD for the former was notably higher than the latter (*P* < 0.05).

**Figure 3:**
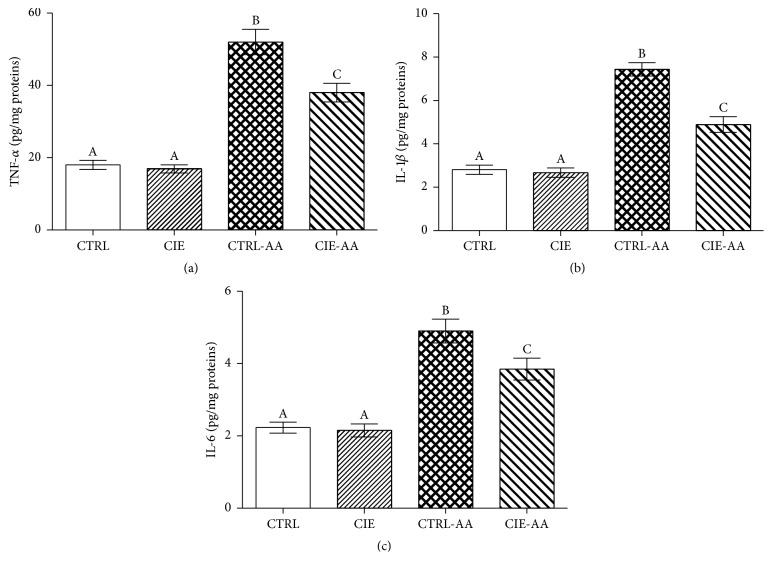
The preventative impact of CIE on the level of (a) TNF-*α*, (b) IL-1*β*, and (c) IL-6 values for the AA model. Data indicated with different superscript letters were statistically dissimilar (*P* < 0.05; *n* = 10).

This study took measures to derive a quantitative measure of the levels for TNF-*α*, IL-1*β*, and IL-6, primarily because this information is key to an accurate understanding of the function that* Chrysanthemum indicum *performed in the experimental rat model.  Figure 3 shows that, for the CTRL and CIE groups, TNF-*α*, IL-1*β*, and IL-6 levels are virtually identical. Dissimilarly, when comparing the CTRL-AA group to the CTRL and CIE groups, it was observed that TNF-*α*, IL-1*β*, and IL-6 levels were greater for the former (*P* < 0.05). Therefore, the results indicate a clear causation between the consumption of CIE for the CIE-AA mice and a fall in TNF-*α*, IL-1*β*, and IL-6 levels (*P* < 0.05).

**Figure 4:**
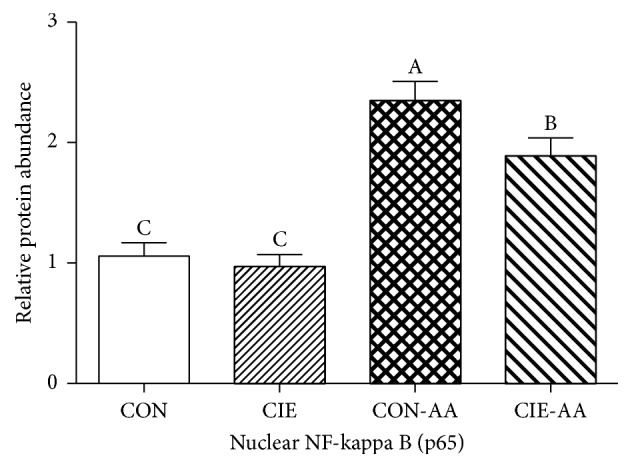
The impact of CIE on NF-*κ*B activation. NF-*κ*b were evaluated by assessing p65 DNA binding in joint. Data indicated with different superscript letters were statistically dissimilar (*P* < 0.05; *n* = 10).

Through the assessment of nuclear NF-*κ*B (p65), it was possible for the researcher to identify NF-*κ*B activation in cell extracts from joint. In turn, this information facilitated the identification of whether suppression of NF-*κ*B activation pathways resulted in the protective impact of isoflavones with regard to arthritis.  Figure 4 illustrates that, for the CTRL-AA and CIE-AA groups, nuclear NF-*κ*B (p65) were considerably greater than in the other two groups (*P* < 0.05). In addition to this, the collected data demonstrated that when comparing the CTRL-AA group with the CIE-AA group, nuclear NF-*κ*B (p65) was considerably greater in the former (*P* < 0.05).

## 4. Discussion

Aside from a recently conducted study, which found evidence to suggest that the butanol-soluble component of* Chrysanthemum indicum* resulted in the inhibition of auricle edema in mice [18], research addressing the anti-inflammatory function of* Chrysanthemum indicum* and, moreover, its molecular mechanism is not extensive. This research demonstrates that* Chrysanthemum indicum* extract was a key contributing factor in facilitating a rise in body weight gain and a reduction in arthritis scores is, therefore, a valuable addition to the extant literature.

Dietary factors play essential roles in body health, disease status, and inflammatory responses [19]. This study has examined the potential that CIE has to play an important role in treating and preventing adjuvant arthritis, and the results are promising regarding its therapeutic role as a therapeutic agent against inflammatory conditions. Lee et al. examined the phagocytic activity of macrophages using a mouse model, and the findings revealed that CIE had a positive impact [20]. Another study, that of Cheng et al., published further findings to support the way in which CIE and its fractions may be beneficial for its anti-inflammatory features; specifically, Cheng et al.'s study addressed mouse auricle edema [18]. Nevertheless, although the extant findings provide insight into the degree to which* Chrysanthemum indicum *has anti-inflammatory properties, information regarding its precise mechanisms in vivo model system is scant. Our previous research showed that CIE had beneficial effects on the inflammation responses and oxidative stress in a ankylosing spondylitis model in mice [21]. However, the extant literature contains no studies which address the anti-inflammatory impacts in an adjuvant arthritis model. Consequently, the present research constitutes the only study published to date on the topic of CIE's anti-inflammatory activity and its action mechanisms among adjuvant arthritis rats.

Adjuvant arthritis is a widely used rodent model in studies addressing rheumatoid arthritis owing to similarity of its pathological characteristics to human RA [22]. As noted in this study, the appearance of pannus formation, inflammatory cells infiltration, cartilage degradation, and bone erosion are core features of adjuvant arthritis, and this constitutes the central rationale as to why adjuvant arthritis is popular in fundamental RA research and anti-RA therapeutic research. The present research has examined the positive impacts that CIE has on oxidative stress in the serum of AA rats, and it is therefore relevant to note that recently published reports have noted the link between RA and oxidative stress in human and animal populations [23, 24]. Furthermore, regarding sufferers of RA, studies have reported on increased lipid peroxidation, oxidative stress, and a decrease in enzymatic antioxidants, such as GSH-Px and SOD [23, 24]. Additional research demonstrates that MPO constitutes a frequent in vivo index of granulocyte infiltration and inflammation and, moreover, it functions as an indicator of oxidative stress [25].

This study found that oxidative stress was higher in the serum of AA rats when considered in relation to the control groups, and a correlation was identified between the presence of dietary CIE and the suppression of MPO activity; the latter finding indicates the alleviation of oxidative stress among AA rats. These findings reinforce the experimental outcomes of Comar et al. [26], which indicate a link between increased ROS content in the liver of arthritic rats and a stimulated prooxidant system in combination with an insufficient antioxidant defence mechanism. In view of the integral part that ROS plays in RA, a connection can be established between the serum's biochemical and histological modifications and variance in the oxidative state. Our previous data also showed that CIE significantly increased the activities of catalase (CAT), SOD, and GSH-Px in ankylosing spondylitis mice [21]. In view of this, alleviating oxidative stress could serve as a viable way to facilitate the prevention and treatment of the liver complications associated with arthritis. By focusing on the serum of arthritic rats, this study also examined the impact that CIE had on the oxidative stress parameters; one of the key findings is that the consumption of CIE among AA rats resulted in higher GSH-Px and SOD activities in the long-term. The author suggests that CIE, owing to its effect of heightening antioxidant enzyme activity, alleviated oxidative stress in the liver.

A series of papers have found evidence for the relevance of NF-*κ*B in RA [27, 28], and the degree to which it is inhibited by CIE has been identified as an indicator of CIE's promise as therapeutic agent. A number of recently conducted experimental models have found that mediators of this kind have the capacity to gather leukocytes, including neutrophils [29, 30]. This study's findings emphasise the potential of CIE insofar as it can facilitate the significant inhibition of inflammatory response and, moreover, decrease NF-*κ*B, TNF-*α*, IL-1*β*, and IL-6 levels. Therefore, there is reason to suppose that the anti-inflammatory capacity of CIE could be applied to inhibit inflammatory mediators including NF-*κ*B, TNF-*α*, IL-1*β*, and IL-6. The same trends were also observed in our previous report; CIE modulated NF-*κ*B pathway and further altered the levels of TNF-*α*, IL-1*β*, and IL-6 [21]. Inflammatory cytokine generation is a critical process in the regulation of inflammation and the advancement of tumours, and this study's findings corroborate CIE's capacity to inhibit TNF-*α* and IL-1*β*. Consequently, the body of evidence to suggest that CIE constitutes an effective therapeutic agent against tumour progression and inflammatory response is growing.

As aforementioned, this study's findings, derived from experimentation with AA rats, demonstrate that CIE improved oxidative stress and, furthermore, facilitated a fall in the serum levels of IL-1*β*, IL-6, and TNF-*α*. An equally critical finding stems from the indication that CIE has the capacity to suppress NF-*κ*B activation in the AA rats' joints. In view of these considerations, CIE may yet emerge as a viable and highly effective way to prevent and treat RA.
